# The Week's Work

**Published:** 1911-04-08

**Authors:** 


### The Week's Work.

### THE ROYAL INFIRMARY, SUNDERLAND.

We are happy to state that the Sunderland Infirmary's petition, recorded in The Hospital of February 11 (page 580), has been answered favourably, and that the institution will henceforward be known as the Royal Infirmary. Mr. R. T. Blumer, chairman of the Finance Committee, and Mr. Thomas Robinson, the Secretary, must take heart of grace and see that the hospital supports its new dignity by carrying out as soon as possible the reconstruction of the old administrative block, so often postponed for lack of funds, and by raising the £2,000 necessary for the opening and maintenance of the Children's Hospital, its daughter institution. Mr. Hamar Greenwood, M.P., who handed the petition to the Home Secretary, has done his part for the governors, and the working-men and subscribers generally now have to do theirs so that the institution may carry its royal privilege worthily by completing the necessary works above mentioned. A great effort made at present would secure the money needed, for local support could receive no stronger stimulus than his Majesty's approval of the work already done.

### HOSPITALS AND OUT-PATIENTS: TWO SUGGESTIONS.

Sir G. H. Kenrick's recent suggestion that "all institutions (in Birmingham) be brought together and opened freely to the whole of the inhabitants of the city, and the whole of the medical men within the city be so employed that their services be properly remunerated," has attracted attention on account of the acuteness of the out-patient problem there. He bases his idea on the Government's proposals for universal insurance against illness and unemployment, and suggests tentatively that a contribution of five shillings be made compulsory within the area of Greater Birmingham. This contribution, which might be deducted from wages, would work out, he thought, at 2d. in the £, and be applicable to all whose incomes were under £150 a year. It will be noticed that in essence this is a financial expedient merely, and, though the control of all the city hospitals under a "strong board" is suggested, no attempt to deal with the out-patient problem from a national point of view has been defined. As our readers will remember, in Sir Henry Burdett's pamphlet, "The Future of the Hospitals," the question is attacked on wider ground. There the patient, the practitioner, and the State are considered in correlation, and the abolition of the out-patient department is the first recommendation. The second is its replacement by a casualty department to deal with accidents and urgent cases, and by a sorting-office where all persons applying for treatment would be assigned either to the general practitioner or to the Poor Law, or to the hospital itself, where free treatment, or one on a sliding scale of payment, would be given to them. This plan has the advantage of starting from the patient himself, and, by a simple process of classification, of co-ordinating harmoniously the services of the practitioner, the voluntary hospital, and the Poor Law. It also discusses proposals for a State insurance scheme. We do not believe that any scheme can be successful which does not combine these three agents, and none must start simply with the object of raising money to support the want of combination which at present prevails.

### THE CHOICE OF A HOSPITAL SECRETARY.

Whenever a vacancy occurs in the post of secretary at a large hospital, the question still arises, "What sort of man shall we have?" It remains an open question; it is still undecided what qualities are necessary for the post. In the old days it was often given to a man who had been pensioned for his successful work at something else. To-day it may be given almost to any type of man, so varied are its duties and so varied are the policies which different institutions adopt. The two articles recently published in The Hospital make this clear. One advocated a journalistic, the other a business type. At present, it is true, a hospital usually inclines to one of these. There is a third type, however, which is something of a survival; it may be called the figure-head. "Give us," say its advocates, "a man as secretary whose name or position is known to the public, and his administrative duties can be performed by his clerks." But, it may be asked, can the work of a hospital secretary be divided into routine on the one hand and publicity on the other? The value of the figure-head in other positions is obvious: in this it has generally meant that the organiser was present, and the work done under another name. Diversity of view is bound to result when each hospital has to work out its own solvency, but the raising of money is only one of a hospital secretary's duties, as is recognised by those, for instance, who believe in the medical superintendent.

### THIS WEEK'S DRUG MARKET.

Business continues quiet, but most of the price-changes are in an upward direction. Some of the makers of cocaine are quoting higher prices. Siam gum benzoin is extremely scarce, and a further advance on the already high prices is expected. Oil of almonds is slightly dearer. Balsam of Peru is dearer, with a probability of a further advance. The price of jalap is rising, owing to a shortage of crop. Pareira root is dearer. Higher prices are quoted for gentian root. The demand for cod-liver oil is very quiet, as buyers are unwilling to make purchases at the extreme figures asked. Calomel, corrosive sublimate, and other salts of mercury are slightly cheaper, in consequence of the fall in the value of quicksilver. Tartaric acid is rather lower, but may be advanced again. Cream of tartar is also rather lower. Ergot of rye is tending somewhat cheaper. The position of opium is still unchanged.

